# From Brain Structure to Stress Response: COVID‐Specific Anxiety Links Pre‐Pandemic Gray Matter Volume to Changes in Life Satisfaction

**DOI:** 10.1155/da/7767487

**Published:** 2026-10-29

**Authors:** Zhuqing Feng, Graham J. Kemp, Song Wang

**Affiliations:** ^1^ Department of Radiology West China Hospital Sichuan University Chengdu China scu.edu.cn; ^2^ College of Teacher Education Sichuan University of Arts and Science Dazhou China sasu.cn; ^3^ Institute of Life Course and Medical Sciences University of Liverpool Liverpool UK liv.ac.uk

**Keywords:** anxiety, brain structure, COVID-19 pandemic, life satisfaction, voxel-based morphometry

## Abstract

The COVID‐19 pandemic severely threatened mental well‐being, potentially impacting individual life satisfaction (LS); thus, this study sought to identify pre‐pandemic brain structural markers associated with subsequent LS changes and explore the role of COVID‐specific anxiety in this relationship. We utilized structural magnetic resonance imaging (MRI) and behavioral data (including the LS scale) collected before the pandemic (T1) from 115 general university students, followed up 1 year after the outbreak (T2) for LS and COVID‐anxiety measures. Employing whole‐brain voxel‐based morphometry (VBM) and confirmatory prediction analyses, we identified brain regions where pre‐pandemic gray matter volume (GMV) correlated with subsequent LS changes (ΔLS from T1 to T2), followed by mediation analysis to assess the indirect effect of COVID‐anxiety. While group‐level ΔLS was stable, individual variability showed a significant negative correlation with pre‐pandemic GMV in the pregenual anterior cingulate cortex (pgACC) and left superior temporal gyrus (STG), and a positive correlation with GMV in right precuneus. Furthermore, COVID‐anxiety correlated with ΔLS, and mediation analysis revealed a significant negative indirect effect of COVID‐anxiety on the link between pgACC GMV and ΔLS, such that greater pre‐pandemic pgACC GMV was associated with higher COVID‐specific anxiety, which in turn was associated with a decrease in LS. This mediation effect was partial, as the direct path from pgACC GMV to ΔLS remained significant. Crucially, these results were independent of socioeconomic status, stressful life events, and general mental health. Overall, our study indicates that pre‐pandemic brain structure can prospectively predict individual LS changes during the pandemic, and this relationship was mediated by COVID‐specific anxiety. These findings shed light on the neuroanatomical basis of LS and could have implications for targeted interventions during public health crises and similar challenges.

## 1. Introduction

The global pandemic of coronavirus disease 2019 (COVID‐19) has posed a major threat to public health and well‐being, not just because of its biological pathogenicity but also for the strict lockdowns and quarantines imposed to control its spread [1]. Faced with unknown or poorly understood risks, people are prone to fear and nervousness, with consequent effects on subjective well‐being [2, 3]. Especially in individuals with coping difficulties, this may lead to mental health problems such as anxiety, depression, and posttraumatic stress disorders (PTSDs) [4]. One way to capture the complexity of psychological and behavioral responses during the pandemic is life satisfaction (LS). This measure of subjective well‐being and mental health is based on a person’s cognitive evaluation of their overall satisfaction with their life situation [2, 3]. In cross‐sectional studies of the general population during the pandemic, LS remained moderate but lower than pre‐pandemic levels [5–8]. Longitudinal studies reported that LS was unchanged at the start of the epidemic [9], slightly decreased in the early and mid‐term [9–12], and not altered in the long term [13–15]. Potential pandemic‐related causes of low LS include social isolation, fear, and other negative emotional experiences, exacerbated by ineffective coping behaviors [16].

Recent advances in neuroimaging, especially magnetic resonance imaging (MRI), enable researchers to precisely link individual brain differences to LS. Particularly, structural MRI (sMRI) studies in healthy individuals have reported that LS is associated with structural brain features (e.g., gray matter volume [GMV], cortical thickness, and cortical surface area) in multiple brain regions (e.g., superior and middle frontal gyrus [SFG/MFG], ventromedial prefrontal cortex/anterior cingulate cortex [VMPFC/ACC], superior temporal gyrus [STG], hippocampus/parahippocampal gyrus, insula, and precuneus) involved in emotional and social cognitive processing (see two systematic reviews: [17, 18]). No studies to date have examined the neural correlates of LS during the COVID‐19 pandemic, but there is some evidence that pre‐COVID brain signatures can predict psychological well‐being [19, 20], general distress [21, 22], and trauma‐related symptoms [23, 24] during the pandemic. These psychological dimensions are conceptually related to LS, and the reported predictive brain regions overlap with the neural substrates of LS (e.g., SFG/MFG, VMPFC/ACC, STG, insula, hippocampus, and precuneus; see a systematic review: [25]). Bringing these lines of evidence together, the first aim of the present study was to identify the prospective neurostructural markers of LS changes during the pandemic.

An important negative emotional factor detrimental to LS is anxiety [26], which is particularly evident during the pandemic [27]. It has been shown that brain regions engaged in anxiety processing overlap with those concerned with LS, such as SFG/MFG, VMPFC/ACC, insula, and hippocampus [28, 29]. In particular, evidence from two sMRI studies revealed that the pre‐pandemic cortical thickness in the lateral PFC [19] and insula [30] can prospectively predict anxious symptoms during the pandemic. Another resting‐state functional MRI (rs‐fMRI) study found that COVID‐specific anxiety could be predicted by the brain functional connectome, in which the clusters with highest node strength (e.g., SFG/MFG, ACC, insula, STG, precuneus, and hippocampus) also corresponded to the neural substrates of LS [31]. Our second aim was, therefore, to explore whether pandemic‐related anxiety was a mediator between the brain structure and LS changes during the pandemic.

To answer these questions, we exploited pre‐pandemic sMRI data to predict the change of LS before and after the pandemic outbreak in a sample of general university students. Before the COVID‐19 pandemic (from October 2019 to January 2020), we had performed sMRI and a range of behavioral assessments, including measure of LS. After the pandemic outbreak, these participants experienced strict lockdown measures for around 1 year (February 2020 to March 2021), after which (March to April 2021) follow‐up surveys were carried out for LS assessment and additional COVID‐related behavioral measures. Change in LS was assessed as the difference between the two LS tests (T2–T1). As GMV is widely used in personality neuroscience as a neuroanatomical metric to explore the biomarkers of behavioral constructs [32, 33], we used voxel‐based morphometry (VBM) [34] to investigate the association between regional GMV and LS changes. VBM was used to calculate the voxelwise GMV, and then whole‐brain regression analyses identified the clusters whose GMV was significantly related to LS changes. Considering the prior reported brain structural substrates of LS [17, 18], our Hypothesis I was that the GMV in regions of emotion and social cognition processing circuitry (e.g., SFG/MFG, VMPFC/ACC, STG, insula, hippocampus, and precuneus) would significantly predict the subsequent changes of LS. Given the overlap of these regions with those involved in COVID‐related anxiety [19, 25, 30, 31], our Hypothesis II was that the COVID‐anxiety would, in mediation analysis, be a significant link in the association between brain GMV and changes in LS.

## 2. Methods

### 2.1. Participants and Procedures

A total of 151 university students without any history of psychiatric and neurological illnesses were recruited as part of a larger project exploring the neurobiological basis of personality and mental health in the general population [24, 35]. All participants underwent MRI scanning and answered behavioral measures before the COVID‐19 outbreak in China (T1, October 2019–January 2020). One year after the initial outbreak (T2, March–April 2021), the participants were re‐contacted to take another online survey including a repeat LS assessment and additional COVID‐related tests. A total of 127 participants completed the online survey at T2. Of the 127 participants who completed the T2 online survey, 12 were excluded from the final analyses for the following reasons: missing or incomplete T1 behavioral data (*n* = 3), missing or incomplete T2 behavioral data (*n* = 4), and T1 structural images that failed quality control (*n* = 5). Image quality control criteria included (i) no visible artifacts or anatomical abnormalities; (ii) successful segmentation into gray matter, white matter, and cerebrospinal fluid; (iii) independent visual inspection by two experienced neuroimaging researchers. The final sample therefore comprised 115 participants (66 females; mean age = 22.37 ± 2.08 years). None of the participants were infected with COVID‐19, proved by negative nucleic acid tests at both points, and all were right‐handed [36]. The Medical Research Ethics Committee at the West China Hospital of Sichuan University approved the study. Written informed consent was obtained from all participants.

A *post hoc* power analysis was conducted using 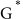
Power 3.1 [23, 35]. For the whole‐brain VBM regression with 115 participants and four predictors (voxelwise GMV, age, sex, and total GMV), the achieved power to detect a medium effect size (*f* ^2^ = 0.15) at *α* = 0.05 was 0.92. For the mediation model, with 115 participants and 5000 bootstrap resamples, the achieved power to detect the observed indirect effect was adequate; sensitivity analysis indicated that the minimum detectable effect size for the indirect path at 80% power was small‐to‐moderate. These results suggest that the final sample size provided sufficient power for the main analyses.

### 2.2. Behavioral Measurements

The Satisfaction With Life Scale (SWLS), a standard instrument [37], was used to measure LS at T1 and T2. This consists of five entries, such as “In most ways my life is close to my ideal,” with 7‐point Likert scoring of agreement or disagreement, a higher total score indicating greater LS. The Chinese version of the SWLS is a reliable and effective tool [38, 39]; Cronbach’s *α* was 0.81 and 0.88 at T1 and T2, respectively, indicating sufficient internal reliability.

Two scales reflecting pandemic‐related anxiety were used at T2 to examine links between the COVID‐anxiety and LS changes. The COVID‐19 Anxiety Syndrome Scale (C‐19ASS) [40] has nine items, divided into two dimensions (perseverate thinking and avoidance), and participants report the frequency of each over the previous 2 weeks with a 5‐point Likert scale (from 1 = not at all to 5 = nearly every day), a higher total score indicating more severe COVID‐19‐related anxiety. This scale has been translated into Chinese and is widely used to assess anxiety symptoms in Chinese populations [41, 42]. Cronbach’s *α* for the C‐19ASS in this study was 0.89, showing good internal reliability. To quantify the anxiety‐related reassurance‐seeking behavior, we used the Coronavirus Reassurance‐Seeking Behaviors Scale (CRSBS) [43]. This has five items, and participants report the frequency of each over the previous 2 weeks with a 5‐point Likert scale (from 1 = not at all to 5 = nearly every day), with a higher total score indicating more intense reassurance‐seeking. Adhering to Hambleton’s guidelines for test translation and adaptation [44], the original English items of the CRSBS were first translated into Chinese by one translator, followed by a back‐translation into English by a second translator to ensure conceptual equivalence and accuracy. Cronbach’s *α* for the CRSBS in this study was 0.86, indicating good internal reliability.

Since common stressful life events, psychological health conditions, and socioeconomic status may affect the associations between brain structure, LS, and COVID‐anxiety, three scales measured at T1 were used as additional controlling measures: the Self‐Rating Life Events Checklist (SRLEC) [45], the General Health Questionnaire‐12 (GHQ‐12) [46], and the Socioeconomic Status Scale (SSS) [47]. These measures have been widely applied to various Chinese populations and have demonstrated satisfactory psychometric properties [22, 24, 35]. In this study, the Cronbach’s *α* for these measures was 0.90, 0.85, and 0.72, respectively, indicating adequate to good internal reliability.

### 2.3. Neuroimaging Data Acquisition and Preprocessing

Neuroimaging data were acquired using a 3.0T Siemens Trio scanner (Erlangen, Germany) with a 12‐channel head coil at West China Hospital, Chengdu, China. The sMRI scanning used a 3D magnetization‐prepared rapid gradient echo T1‐weighted sequence (repetition time/echo time/inversion time 1900/2.26/900 ms, voxel size 1 × 1 × 1 mm^3^, 176 slices, flip angle 9°, matrix size 256 × 256, and slice thickness 1 mm).

The sMRI data were preprocessed using Statistical Parametric Mapping software (SPM12) [48]. After checking for anatomical abnormalities and artifacts, all brain images were manually reorientated to the anterior commissure, and the segmentation tool in SPM12 was used to segment them into gray matter, white matter, and cerebrospinal fluid. Using SPM12‐Diffeomorphic Anatomical Registration Through Exponential Lie Algebra (DARTEL) [49], the GMV data were aligned, resampled to 1.5 × 1.5 × 1.5 mm^3^, registered to the Montreal Neurological Institute (MNI) space, and modulated to preserve the GMV by the inverse Jacobian of the local transformations. The normalized modulated data were smoothed with an 8 mm full‐width at half‐maximum Gaussian kernel and quality‐checked independently by two experienced neuro‐radiological researchers.

### 2.4. VBM Analysis

Whole‐brain VBM in SPM12 was used to build a linear regression model to locate the brain regions specifically related to LS changes between T1 and T2: the dependent variable (*Y*) was the alteration in LS (ΔLS = SWLS at T2 − SWLS at T1, such that positive values indicate an increase in LS and negative values indicate a decrease in LS), the voxelwise GMV was the independent variable (*X*), and age, sex, and total GMV were included as covariates. To correct for multiple comparisons, random field theory was used to extract significant clusters of voxels at the familywise error rate of *p* < 0.05 (for a *p* voxel threshold <0.001), by modeling noise as a Gaussian random field both for peaks and spatial extent [23, 35, 50].

### 2.5. Confirmatory Prediction Analysis

Balanced cross‐validation machine learning was used to investigate the stability of the association between ΔLS and the pre‐pandemic GMV of the clusters identified in whole‐brain VBM analysis [23, 39, 51, 52]. A fourfold balanced cross‐validation was performed using linear regression (with ΔLS = *Y* and GMV = *X*) to calculate the predictive ability [*r*_(predicted, observed)_] of GMV for ΔLS: the dataset was separated into four units (to ensure balanced distributions of all variables across the units), and three of which were selected to establish a linear regression for predicting the data in the fourth; by doing this for all four selections, we obtained an ultimate *r*_(predicted, observed)_ characterizing the correlation between the observed and predicted model data. Nonparametric testing was used to estimate the statistical significance of *r*_(predicted, observed)_ using a repeated process of random rearrangement of the *X* data to calculate *r*_(predicted, observed)_ with the original *Y* [23, 39]. Under the null hypothesis of no relationship between ΔLS and GMV, a null distribution of *r*_(predicted, observed)_ was generated by repeating 5000 times: one minus the percentile of the true *r*_(predicted, observed)_ was taken as the final *p* value. The analysis was controlled for age, sex, and total GMV.

### 2.6. Mediation Analysis

To explore the indirect effect of brain structure on LS changes through COVID‐anxiety, a mediation model was tested by a bootstrapping approach using the macro PROCESS in SPSS software [53]. Regional LS‐related GMV was treated as X, COVID‐anxiety as the mediator variable (*M*), ΔLS as the *Y*, and sex, age, and total GMV as the control variables. According to the standard algorithm, the mediating effect is analyzed via four paths: path *c* (the relation of *X* and *Y*), path *c*′ (the relation between *X* and *Y* after adjusting for *M*), path *a* (the relation of *X* and *M*), and path *b* (the relation of *M* and *Y* after adjusting for *X*). The mediating effect is estimated as the value of (*c*–*c*′) or (*a* × *b*), and its statistical significance is determined from the bootstrapped 95% confidence intervals (CIs) after 5000 iterations (not including zero).

## 3. Results

### 3.1. Behavioral Measures

Table 1 lists the descriptive statistics of the demographic data and behavioral correlations. A paired‐sample *t*‐test at T1 and T2 showed that LS did not change significantly (*t* = 1.68, *p* = 0.095) at the group level: individually, just over half (55%) of the participants showed an increase (*N* = 63, ΔLS [mean ± standard deviation] = 4.02 ± 2.51), about a third (34%) showed a decrease (*N* = 39, ΔLS = −4.36 ± 2.93), and a few (11%) did not change (*N* = 13, ΔLS = 0). Across the whole group, individual ΔLS was negatively correlated with the scores of C‐19ASS (*r* = −0.19, *p* = 0.043) and CRSBS (*r* = −0.28, *p* = 0.003), but not with age (*r* = 0.04, *p* = 0.647), sex (*r* = 0.07, *p* = 0.479), or total GMV (*r* = −0.17, *p* = 0.077). C‐19ASS and CRSBS scores showed a significant large positive correlation (*r* = 0.72, *p* < 0.001), suggesting that they may measure a similar construct of COVID‐anxiety [54]. We, therefore, assigned equal weight to the normalized raw scores of C‐19ASS and CRSBS and calculated their average *Z* scores as a new metric to measure COVID‐anxiety in the subsequent analyses [55]. This score showed a significant negative association with ΔLS (*r* = −0.26, *p* = 0.005).

**Table 1:** Demographic information and bivariate correlations.

Variable	Mean	SD	1	2	3	4	5	6	7	8	9	10
1. Sex^a^ (T1)	—	—	—	—	—	—	—	—	—	—	—	—
2. Age (T1)	22.37	2.08	−0.10	—	—	—	—	—	—	—	—	—
3. SSS (T1)	4.95	1.49	0.02	0.05	—	—	—	—	—	—	—	—
4. SRLEC‐frequency (T1)	12.23	5.78	−0.04	−0.06	−0.22 ^∗^	—	—	—	—	—	—	—
5. SRLEC‐impact (T1)	28.11	16.66	0.04	−0.02	−0.20 ^∗^	0.93 ^∗∗∗^	—	—	—	—	—	—
6. GHQ‐12 (T1)	19.34	4.64	0.16	0.11	−0.26 ^∗∗^	0.28 ^∗∗^	0.29 ^∗∗^	—	—	—	—	—
7. SWLS (T1)	20.36	5.40	0.09	−0.05	0.47 ^∗∗∗^	−0.25 ^∗∗^	−0.25 ^∗∗^	−0.35 ^∗∗∗^	—	—	—	—
8. C‐19ASS (T2)	13.50	4.43	−0.01	0.07	−0.07	0.21 ^∗^	0.24 ^∗∗^	0.12	0.03	—	—	—
9. CRSBS (T2)	7.21	2.70	−0.03	0.10	−0.00	0.15	0.16	0.12	0.12	0.72 ^∗∗∗^	—	—
10. SWLS (T2)	21.08	6.55	0.12	−0.01	0.50 ^∗∗∗^	−0.19 ^∗^	−0.17	−0.28 ^∗∗^	0.72 ^∗∗∗^	−0.11	−0.10	—
11. ΔLS (T2‐T1)	0.72	4.60	0.07	0.04	0.16	0.03	0.05	0.01	−0.15	−0.19 ^∗^	−0.28 ^∗∗^	0.58 ^∗∗∗^

*Note:* ΔLS, SWLS at T2 − SWLS at T1; T1: October 2019–January 2020; T2: March–April 2021.

Abbreviations: C‐19ASS, COVID‐19 Anxiety Syndrome Scale; CRSBS, Coronavirus Reassurance‐Seeking Behaviors Scale; GHQ‐12, General Health Questionnaire‐12; SD, standard deviation; SRLEC, Self‐Rating Life Events Checklist; SSS, Socioeconomic Status Scale; SWLS, Satisfaction With Life Scale.

^a^Male 0, Female 1.

^∗∗∗^ *p* < 0.001.

^∗∗^ *p* < 0.01.

^∗^ *p* < 0.05.

### 3.2. Pre‐COVID GMV Markers of Pandemic‐Related LS Changes

In whole‐brain VBM analysis, ΔLS was negatively associated with pre‐pandemic GMV in pregenual ACC (pgACC; *r* = −0.32, *p* < 0.001) and left STG (*r* = −0.35, *p* < 0.001; Table 2 and Figure 1), and positively associated with GMV in right precuneus (*r* = 0.41, *p* < 0.001; Table 2 and Figure 2). We selected the pgACC, left STG, and right precuneus as regions of interest (ROIs) for machine learning predictive analyses: ΔLS was significantly predicted by pre‐pandemic GMV of pgACC [*r*_(predicted, observed)_ = 0.28, *p* < 0.001], left STG [*r*_(predicted, observed)_ = 0.31, *p* < 0.001], and right precuneus [*r*_(predicted, observed)_ = 0.38, *p* < 0.001], controlling for age, sex, and total GMV.

**Figure 1:**
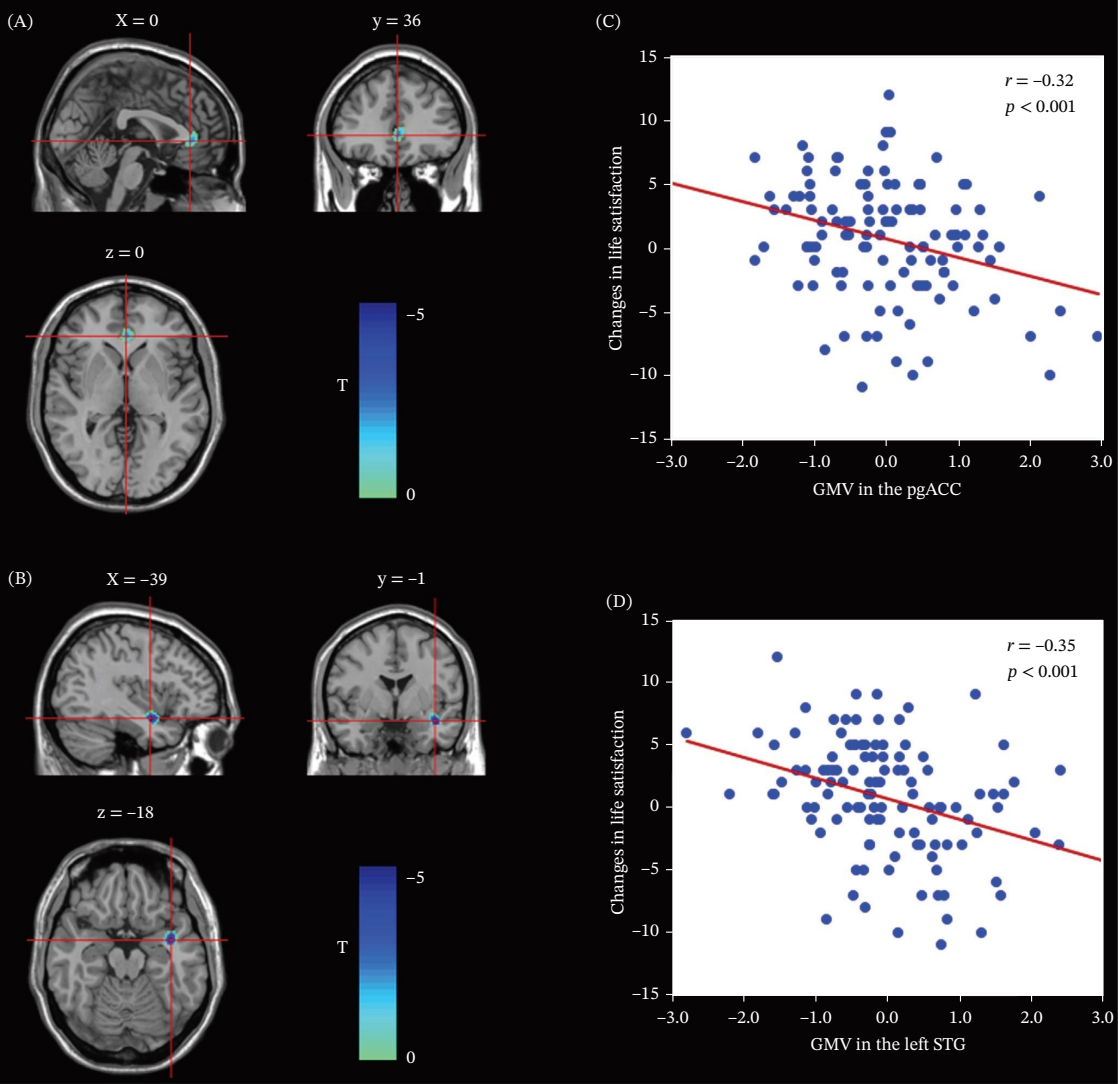
Brain regions showing negative association with life satisfaction changes. Brain images show that GMV in the pgACC (A) and left STG (B) was negatively related to life satisfaction changes. Scatter plots depicting the relationship between life satisfaction changes and GMV in the pgACC (C) and left STG (D). The scores on the *y*‐axis represent the raw value of life satisfaction changes (ΔLS, SWLS at T2 − SWLS at T1; positive values indicate an increase in life satisfaction). The scores on the *x*‐axis represent the standardized residuals of the GMV after sex, age, and total GMV were regressed out. GMV, gray matter volume; pgACC, pregenual anterior cingulate cortex; STG, superior temporal gyrus.

**Figure 2:**
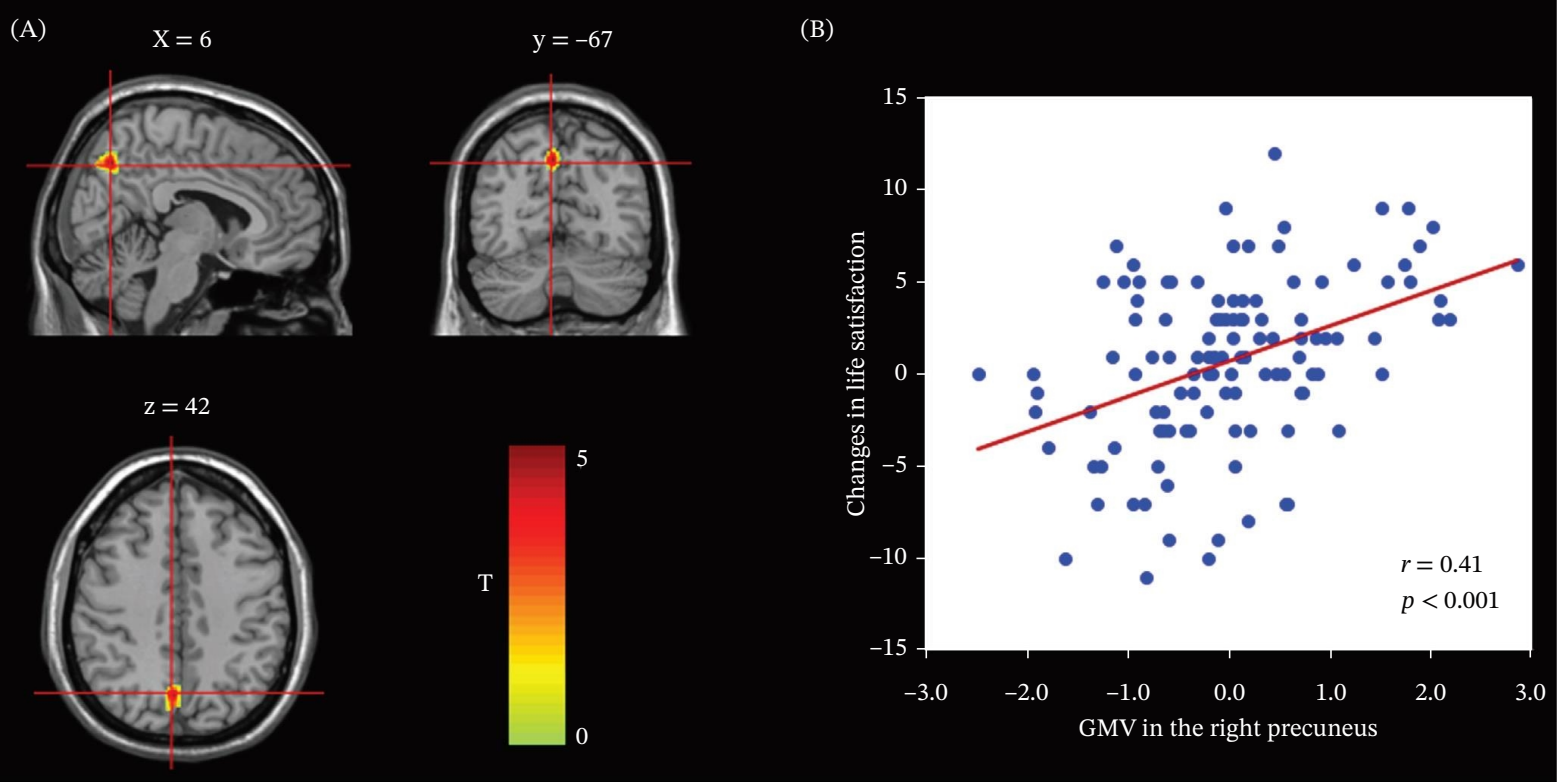
Brain regions showing positive association with life satisfaction changes. Brain images show that GMV in the right precuneus (A) was positively related to life satisfaction changes. Scatter plots depicting the relationship between life satisfaction changes and GMV in the right precuneus (B). The scores on the *y*‐axis represent the raw value of life satisfaction changes (ΔLS, SWLS at T2 − SWLS at T1; positive values indicate an increase in life satisfaction). The scores on the *x*‐axis represent the standardized residuals of the GMV after sex, age, and total GMV were regressed out. GMV, gray matter volume.

**Table 2:** Brain regions where gray matter volume linked with life satisfaction changes.

Region	BA	MNI coordinate			Peak *T* score	Cluster size (voxels)
		*x*	*y*	*z*		
Negative correlation						
Bilateral pgACC	32	0	36	0	−3.60	300
Left STG	22	−39	−1	−18	−4.42	299
Positive correlation						
Right precuneus	7	6	−67	42	5.10	371

*Note:* Threshold for significant regions: *p* < 0.001 at voxel level and *p* < 0.05 at cluster level (Gaussian random field correction approach).

Abbreviations: BA, Brodmann’s area; MNI, Montreal Neurological Institute; pgACC, pregenual anterior cingulate cortex; STG, superior temporal gyrus.

### 3.3. COVID‐Anxiety Links GMV to Pandemic‐Related LS Changes

To test whether COVID‐anxiety mediates the GMV‐ΔLS association, we first tested whether it was linked to GMV in the clusters already shown to be associated with ΔLS. Only the GMV of pgACC was significantly associated with COVID‐anxiety (*r* = 0.23, *p* = 0.015), the associations of left STG (*r* = 0.15, *p* = 0.106) and right precuneus (*r* = −0.11, *p* = 0.258) being nonsignificant. The association with pgACC survived correction for age, sex and total GMV (*r* = 0.23, *p* = 0.016), while the associations with left STG (*r* = 0.15, *p* = 0.111) and right precuneus (*r* = −0.11, *p* = 0.264) remained nonsignificant. Then, we established and validated the mediation model of “pgACC (T1)—COVID‐anxiety (T2)—ΔLS (T2 − T1)” with the PROCESS approach in SPSS. As hypothesized, COVID‐anxiety partially mediated the relationship between the pre‐COVID GMV of pgACC and ΔLS (Figure 3). The path coefficients were as follows: path *a* (pgACC GMV → COVID‐anxiety) = 0.23, *p* < 0.05; path *b* (COVID‐anxiety → ΔLS, adjusting for pgACC GMV) = −0.20, *p* < 0.05; total effect path *c* (pgACC GMV → ΔLS) = −0.32, *p* < 0.001; direct effect path *c*′ (pgACC GMV → ΔLS, adjusting for COVID‐anxiety) = −0.27, *p* < 0.01. The indirect effect was significant (effect size: −0.05, 95% CI: [−0.10, −0.01], *p* < 0.05), indicating that COVID‐anxiety mediated the association between pgACC GMV and ΔLS. Because path *c*′ remained significant after adjusting for the mediator, mediation was partial rather than full. This mediation effect survived controlling for age, sex and total GMV (effect size: −0.05, 95% CI: [−0.10, −0.01], *p* < 0.05).

**Figure 3:**
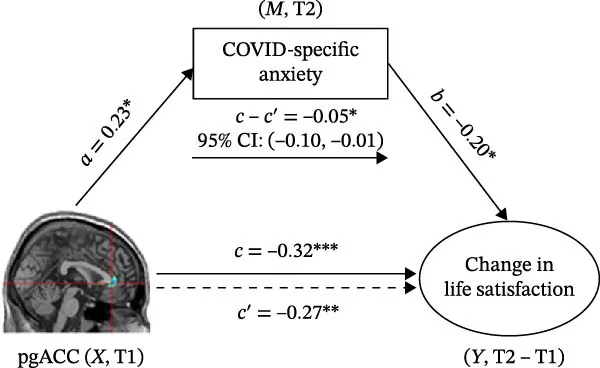
Mediating role of COVID‐specific anxiety in the link between pgACC volume and life satisfaction changes. Path coefficients are standardized (*a* = 0.23 ^∗^; *b* = −0.20 ^∗^; *c* = −0.32 ^∗∗∗^; *c*′ = −0.27 ^∗∗^). Because *c*′ remained significant, the mediation was partial. Age, sex, and total GMV were adjusted in the mediation model. CI, confidence interval; *Y*, dependent variable; *X*, independent variable; *M*, mediator variable; pgACC, pregenual anterior cingulate cortex; T1, from October 2019 to January 2020; T2, from March to April 2021.  ^∗∗∗^ *p* < 0.001;  ^∗∗^ *p* < 0.01;  ^∗^ *p* < 0.05.

### 3.4. Specificity of the Findings

To test the specificity of COVID‐anxiety, we added SSS, GHQ‐12, and SRLEC scores as additional confounding variables. ΔLS was still negatively correlated with COVID‐anxiety (*r* = −0.28, *p* = 0.005), and also with GMV in pgACC (*r* = −0.32, *p* = 0.001), left STG (*r* = −0.35, *p* < 0.001), and right precuneus (*r* = 0.42, *p* < 0.001). In mediation analysis, the model of “pgACC (T1)—COVID‐anxiety (T2)—ΔLS (T2 − T1)” was still significant (effect size = −0.04, 95% CI: [−0.09, −0.01], *p* < 0.05).

## 4. Discussion

This study investigated the power of pre‐pandemic brain structure to predict individual LS changes during the COVID‐19 pandemic and the mediating effects of COVID‐anxiety in this relationship. The results showed that the pre‐pandemic GMV of pgACC and left STG was negatively related to the LS alterations of individuals, while GMV in right precuneus was positively correlated with LS changes. Moreover, COVID‐anxiety partially mediated the association between GMV in pgACC and LS changes. These findings persisted robustly even after controlling for family socioeconomic status, other stressful events, and general mental health in the statistical analyses.

LS is usually regarded as a fairly stable property, relatively insensitive to environmental influences [56, 57]. Nevertheless, the majority of our subjects did show LS change up or down. However, because similar numbers of subjects showed positive and negative changes, the overall effect was not significant at the group level, as in other studies of healthy adults in the pandemic [13–15]. The question addressed in this study was to what extent this individual variability is related to pre‐pandemic brain structure, specifically (our Hypothesis I), the GMV in regions of the emotion and social cognition processing circuitry. We did indeed find that GMV in three such regions (pgACC, left STG, and right precuneus) could predict individual LS changes. Furthermore, COVID‐anxiety partially mediated the association between LS changes and GMV in pgACC (in accordance with our Hypothesis II). Taken together, our study provides preliminary evidence indicating that pre‐pandemic brain GMV can predict individual LS alterations during the epidemic and highlights COVID‐anxiety as a potential mediator in this relationship. We now discuss their possible pathophysiological implications.

### 4.1. Brain Regions Linked to LS Changes

GMV in pgACC was negatively associated with ΔLS. Other negative relationships have been reported between the brain structure and positive mental constructs [35, 39, 58], generally explained in terms of intracortical myelinization and synaptic pruning during development [59]. The pgACC connects prefrontal regions to the limbic system, delivering cognitive information to emotion‐generation systems [60]. The pgACC is involved in cost–benefit appraisal and decision‐making, of crucial importance in self‐evaluation and reward circuits [61] and active in self‐evaluation of subjective well‐being [62]. Impaired synaptic pruning resulting from stress exposure in adolescence can impair the optimization of prefrontal‐limbic circuitry [63, 64]. At the other end of the spectrum, one might speculate that lower GMV in pgACC reflects successful pruning of ineffective synapses, improving communication between prefrontal and limbic lobe, and so facilitating cognitive‐emotional interactions to enable improvement of LS [65]. Of course, the pandemic itself may be a sufficient adverse experience to have detrimental effects on the connectivity between pgACC and limbic regions [25], but our experimental design could not evaluate this.

GMV in the left STG was also negatively associated with ΔLS. The STG is a core node in social cognition, social perception, and emotional recognition [66] and is implicated in self‐related cognitive processing, such as self‐recognition and self‐monitoring [67, 68], important in the development of adaptive social and self‐regulated behaviors. In healthy young adults, spontaneous neural activity in STG is positively associated with individual differences in cognitive well‐being (i.e., LS [69]) and eudaimonic well‐being [70]. Notably, a relationship between increased GMV in STG and negative emotions in healthy individuals [71] extends to subclinical and clinical symptoms of depression and anxiety [72–74]. It may be that individuals with lower STG GMV adopt a more appropriate coping style, with less negative emotional arousal.

GMV in the precuneus was positively associated with ΔLS. This finding is consistent with previous reports of an association between precuneus GMV and LS [75], subjective happiness [76], and eudaimonic and hedonic balance [77]. The precuneus is a neural substrate of self‐efficacy [78] and self‐consciousness [79] and is involved in various cognitive functions, such as exploratory information‐seeking, autobiographical memory retrieval, and self‐face prioritization [80]. These functions are all important for self‐cognitive behaviors that are crucial for mental health [81]. An increase in precuneus GMV is correlated with successful emotion management, encompassing emotional conflict resolution and positive emotional intensity [76]. Patients with emotional disturbance disorders (e.g., PTSD and bipolar depression) show structural impairment in the precuneus [82, 83]. Thus, a higher pre‐pandemic precuneus GMV may reflect greater proficiency in self‐cognitive appraisal and emotional regulation, which may aid in managing the negative mood states induced by the pandemic.

### 4.2. Mediating Effect of COVID‐Anxiety

Our Hypothesis II was that the COVID‐anxiety would, in mediation analysis, be a significant link in the association between brain structure and changes in LS. This proved to be the case: COVID‐anxiety was negatively associated with LS changes, independent of age, sex, and the scores of SSS, GHQ‐12, and SRLEC, suggesting a specific response to the stressor of the pandemic. This result is consistent with previous reports that higher anxiety tends to be accompanied by greater reductions in LS during the pandemic [8, 12]. Other studies have demonstrated the adverse impact of COVID‐19 events on LS, potentially stemming from poor reactions (e.g., passive coping styles) to negative emotional experiences such as anxiety and depression [84, 85]. The current finding may have implication for targeting anxiety in mitigating the effects of a major public health crisis on LS.

On the brain level, we found that only the pgACC GMV showed a positive correlation with COVID‐anxiety. The pgACC has a pivotal role in the emotion processing network, especially in top‐down emotional regulation in the frontal‐limbic system [86]. Individuals with diminished functional connectivity between the pgACC and limbic areas (e.g., amygdala and insula) are more vulnerable to anxiety symptoms [87–89]. The pgACC is also a key node in the adversity processing circuitry, helping judge and regulate adverse factors to adapt to environmental changes [90]. Previous research has found that social adversity experience could increase the risk of having anxiety disorder in adolescents [91]. And individuals with a perception of social adversity events usually show a serious possibility of displaying a high level of anxiety symptoms [92]. Besides, COVID‐19‐related stress (e.g., social isolation) has been demonstrated to be significantly associated with anxiety [93] and may cause neuronal physiology changes (e.g., synaptic densities and protein levels) in prefrontal cortex [94]. In summary, the COVID‐19 pandemic may change the neural structure of pgACC and affect the perception of social stress and anxiety symptoms of individuals, eventually, to influence one’s mental well‐being and LS level.

### 4.3. Limitations of This Study

There are several limitations in this study. First, we only recruited uninfected general college students, which may reduce the generalizability to other groups (e.g., children and the elderly) and clinical patients (e.g., psychiatry disorders and individuals with COVID‐19 infection). College students may differ from the general population in several relevant ways: they tend to be at an age characterized by ongoing neuroplasticity, which may buffer or reshape stress‐related neural and behavioral responses [95]; they often have better digital access and can maintain social connections through online learning and communication during lockdowns [24]; and they may have relatively higher psychological resilience and stronger peer support networks [96]. These factors could attenuate the impact of pandemic‐related stress on LS and limit the generalizability of our findings to more vulnerable populations. Second, the behavioral tests in our study were self‐report scales, which may cause subjective biases [97]. It will be worthwhile to implement multiple objective instruments (e.g., qualitative interviewing and implicit tasks) in future work. Third, our T2 phase was more than a year post‐outbreak; this may have been long enough for the development of coping strategies which partially mitigated negative effects on LS [13]. Fourth, the MRI data were only acquired before the pandemic, so we could not address pandemic‐related structural change (whether as part of LS change or adaptation in mitigation). Finally, we failed to find other predictive value in some regions hypothesized to be associated with LS (e.g., VMPFC and hippocampus), possibly because we only used GMV as a neural indicator; future studies using multimodal MRI techniques would be useful here.

## 5. Conclusions

This study identified a pre‐pandemic neurostructural marker (GMV in the pgACC, left STG, and right precuneus) of LS changes during the pandemic and demonstrated the mediating effect of COVID‐anxiety on the association between GMV and LS changes. These results offer novel evidence on the neuropsychological mechanisms of LS development during the COVID‐19 pandemic, deepening the understanding of the neuroanatomical basis of LS. From a translational perspective, these findings suggest that pre‐pandemic pgACC GMV, in combination with early assessment of COVID‐specific anxiety, might help identify individuals at risk for pandemic‐related declines in LS who could benefit from early anxiety‐focused interventions, such as cognitive–behavioral therapy [98] or neuromodulation targeting prefrontal‐limbic circuits [99]. Such screening and intervention strategies may be particularly valuable during the early phase of public health crises when timely support can mitigate long‐term effects on mental well‐being.

## Author Contributions

**Zhuqing Feng:** investigation, data curation, software, visualization, writing – original draft. **Graham J. Kemp:** validation, methodology, writing – review and editing. **Song Wang:** conceptualization, project administration, investigation, methodology, funding acquisition, resources, writing – original draft, writing – review and editing.

## Funding

This study was supported by the National Natural Science Foundation of China (Grant 32571262) and the Key Research and Development Program of Sichuan Province (Grant 2023YFS0084).

## Ethics Statement

This study was approved by the Medical Research Ethics Committee of the West China Hospital, Sichuan University (Approval Number: 2021‐1216). Written informed consent was obtained from all participants in this study. All methods were implemented based on the relevant guidelines and regulations contained in the Declaration of Helsinki.

## Conflicts of Interest

The authors declare no conflicts of interest.

## Data Availability

The data that support the findings of this study are available from the corresponding author upon reasonable request.
